# Anti-*Candida* Properties of *Gossypium hirsutum* L.: Enhancement of Fungal Growth, Biofilm Production and Antifungal Resistance

**DOI:** 10.3390/pharmaceutics14040698

**Published:** 2022-03-24

**Authors:** Luciene Ferreira de Lima, Jacqueline Cosmo Andrade-Pinheiro, Maria Audilene Freitas, Adriely Idalina da Silva, Victor Juno Alencar Fonseca, Taís Gusmão da Silva, Josefa Carolaine Pereira da Silva, Rosilaine Honorato de Lima, Débora Lima Sales, Rejane Pereira Neves, Edy Sousa de Brito, Paulo Riceli Vasconcelos Ribeiro, Kirley Marques Canuto, Henrique Douglas Melo Coutinho, Abolghasem Siyadatpanah, Bonglee Kim, Maria Flaviana Bezerra Morais-Braga

**Affiliations:** 1 Laboratory of Applied Mycology of Cariri (LMAC), University Regional of Cariri (URCA), Crato 63105-010, Brazil; luciene.ferreira@urca.br (L.F.d.L.); victorjuno5@gmail.com (V.J.A.F.); taisgusmao96@gmail.com (T.G.d.S.); carolaine.pereira@urca.br (J.C.P.d.S.); rosilainehonorato@gmail.com (R.H.d.L.); debora.lima.sales@gmail.com (D.L.S.); flavianamoraisb@yahoo.com.br (M.F.B.M.-B.); 2 Bioassay Laboratory (Labio), Federal University of Cariri (UFCA), Brejo Santo 63260-000, Brazil; jacqueline.andrade@ufca.edu.br; 3 Laboratory of Medical Mycology Sylvio Campos, University Federal of Pernambuco (UFPE), Recife 50670-901, Brazil; audbiologa@hotmail.com (M.A.F.); adryellealvees@gmail.com (A.I.d.S.); rejane.neves@ufpe.br (R.P.N.); 4 Laboratory Multi-User Natural Product Chemistry (LMQPN), Embrapa Agroindústria Tropical, Fortaleza 60511-110, Brazil; edy.brito@embrapa.br (E.S.d.B.); paulo.riceli@embrapa.br (P.R.V.R.); kirley.canuto@embrapa.br (K.M.C.); 5 Laboratory of Microbiology and Molecular Biology (LMBM), University Regional of Cariri (URCA), Crato 63105-010, Brazil; 6 Ferdows School of Paramedical and Health, Birjand University of Medical Sciences, Birjand 9717853577, Iran; 7 Department of Pathology, College of Korean Medicine, Kyung Hee University, Seoul 02447, Korea

**Keywords:** *Malvaceae*, UPLC–QTOF-MS/MS, biofilm eradication, antimicrobial resistance

## Abstract

(1) Background: *Candida* is a genus of yeasts with notable pathogenicity and significant ability to develop antimicrobial resistance. *Gossypium hirsutum* L., a medicinal plant that is traditionally used due to its antimicrobial properties, has demonstrated significant antifungal activity. Therefore, this study investigated the chemical composition and anti-*Candida* effects of aqueous (AELG) and hydroethanolic (HELG) extracts obtained from the leaves of this plant. (2) Methods: The extracts were chemically characterized by UPLC–QTOF-MS/MS, and their anti-*Candida* activities were investigated by analyzing cell viability, biofilm production, morphological transition, and enhancement of antifungal resistance. (3) Results: The UPLC–QTOF-MS/MS analysis revealed the presence of twenty-one compounds in both AELG and HELG, highlighting the predominance of flavonoids. The combination of the extracts with fluconazole significantly reduced its IC_50_ values against *Candida albicans* INCQS 40006, *Candida tropicalis* INCQS 40042, and *C. tropicalis* URM 4262 strains, indicating enhanced antifungal activity. About biofilm production, significant inhibition was observed only for the AELG-treated *C. tropicalis* URM 4262 strain in comparison with the untreated control. Accordingly, this extract showed more significant inhibitory effects on the morphological transition of the INCQS 40006 and URM 4387 strains of *C. albicans* (4) Conclusions: *Gossypium hirsutum* L. presents promising antifungal effects, that may be potentially linked to the combined activity of chemical constituents identified in its extracts.

## 1. Introduction

*Candida* spp. is a commensal yeast normally found in the oral cavity, urogenital system, gastrointestinal tract, as well as in mucous and cutaneous surfaces of healthy individuals [1]. Despite their importance as constituents of the human microbiota, this genus of yeasts is associated with a wide spectrum of human infections with high morbidity and mortality rates, which is at least partially due to the development of resistance to azoles [1,2].

*Candida albicans* and *Candida tropicalis* are commonly isolated from hospitalized patients with a variety of clinical manifestations, ranging from candidiasis to septicemia [3]. As opportunistic pathogens, these species usually cause severe infection in immunocompromised patients, such as those with neutropenia and malignancy [4]. The remarkable pathogenicity of *Candida* species can be attributed to their virulence arsenal, including biofilm production, morphological transition, efflux pump expression [5], epithelial/endothelial adhesion, degrading enzyme secretion, and phenotypic switching [6].

The development of resistance to the most effective antifungals has stimulated the search for new antifungal compounds, both of synthetic and natural origin. In this context, natural product research has identified several compounds with antimicrobial activity, including against clinically important strains of *Candida*, placing medicinal plants as important weapons in the fight against antifungal resistance [7].

The genus *Gossypium* (Malvaceae) is characterized by the presence of chemical compounds such as terpenes, flavonoids, phenols [8,9], and alkaloids [10], which are potentially linked to the significant number of biological activities demonstrated by the genus, including antifungal, antibacterial, antiparasitic, and antiviral, among others [8,11,12,13]. Ethnobotanical studies have demonstrated that the species *Gossypium hirsutum* is traditionally used in the preparation of antiseptic remedies for a vaginal wash in the *Chapada do Araripe* region (Ceará, Brazil) [14]. Accordingly, evidence has indicated that this species presents antifungal activity [15,16,17], especially due to the presence of phenolic compounds such as flavonoids and terpenes [9,18,19]. However, the potential of this species to combat pathogenic *Candida* strains remains to be investigated.

Thus, considering the phytochemical profile of the genus, as well as the traditional use of the species for the treatment of candidiasis, the present study aimed to characterize the chemical profile of *G. hirsutum* aqueous and hydroethanolic extracts and investigate in vitro their effectiveness against the standard and clinical isolates of *Candida albicans* and *Candida tropicalis.*

## 2. Results

### 2.1. UPLC–QTOF-MS/MS Profile of Gossypium hirsutum *L.*

**Figure 1:**
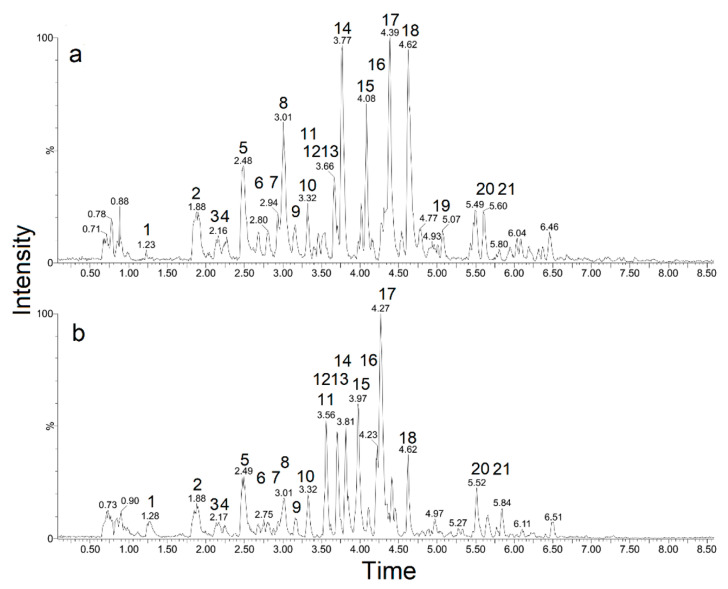
Chemical chromatogram of the (**a**) hydroethanolic (HELG) and (**b**) aqueous (AELG) extracts obtained from *G. hirsutum* leaves.

The UPLC–QTOF-MS/MS analysis of *G. hirsutum* extracts allowed the identification of fifteen constituents, mostly phenolic compounds, as summarized in Figure 1a,b, and Table 1. While the same peaks appeared on both AELG and HELG chromatogram, the latter showed higher relative intensity for the compounds coumaroylglucaric acid, catechin, chlorogenic acid, epi-catechin, while the former showed higher relative intensity for quercetin *O*-hexoside-pentoside.

The compounds **1**, **7**–**11**, **16**, **17**, which showed [M-H]^−^ ion at *m*/*z* 191, 289, 353, 163, 179, 289, and 463, respectively, were identified as citric acid, catechin, chlorogenic acid, *p*-coumaric acid, caffeic acid, epi-catechin, rutin, and quercetin 3-O-glucoside, respectively.

The compounds **2**–**5** showed the same [M-H]^−^ ion at *m*/*z* 371 and exhibited, in MS2, loss of a caffeoyl unit generating glucaric acid (*m*/*z* 209) and glucaric acid (*m*/*z* 191), less H_2_O as product ions. Based on their fragmentation patterns, the compounds are identified as caffeoyl-glucaric acid isomers [18]. Likewise, compound **6** exhibited a [M-H]^−^ ion at *m*/*z* 355 with the same fragmentation pattern at *m*/*z* 209 and *m*/*z* 191, being thus identified as coumaryl-glucaric acid, less one hydroxyl group of caffeoyl-glucaric acid [18].

Compound **15** exhibited a predominant [M-H]^−^ ion at *m*/*z* 595 with a product ion at *m*/*z* 301 and 300, indicating a quercetin aglycone. According to the literature, it was tentatively identified as quercetin O-hexoside-pentoside [18]. Compound **18** showed a predominant [M-H]^−^ ion at *m*/*z* 593 with loss of rutinoside and typical product ions of kaempferol aglycone at *m*/*z* 285 and 255 (loss of HCOH). Based on their fragmentation pattern and literature data, it was identified as kaempferol O-rutinoside.

### 2.2. Anti-Candida Activities

#### 2.2.1. Cell Viability Curve and IC_50_

**Figure 2:**
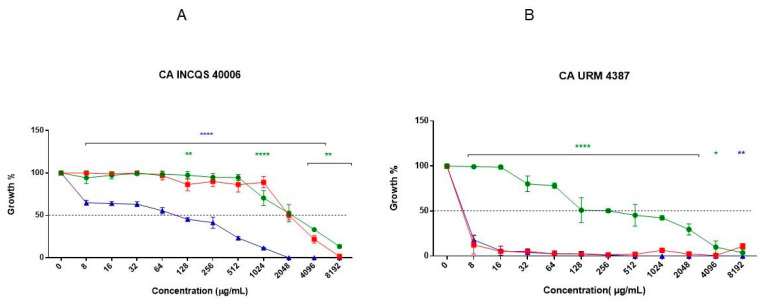

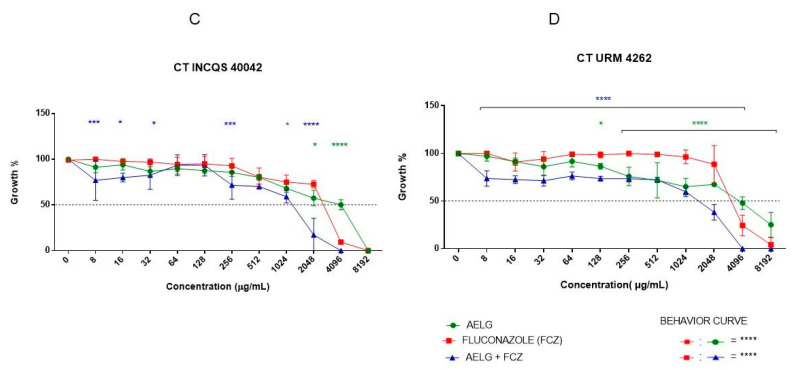
Cell viability curve of AELG alone and in combination with Fluconazole. *Candida* *albicans* INCQS 40006 (**A**), *Candida albicans* URM 4387 (**B**), *Candida tropicalis* INCQS 40042 (**C**) and *Candida tropicalis* URM 4262 (**D**). Statistical significance was determined considering the difference in relation to fluconazole, **** = *p* < 0.0001; *** = *p* < 0.001; ** = *p* < 0.01; * = *p* < 0.1.

**Figure 3:**
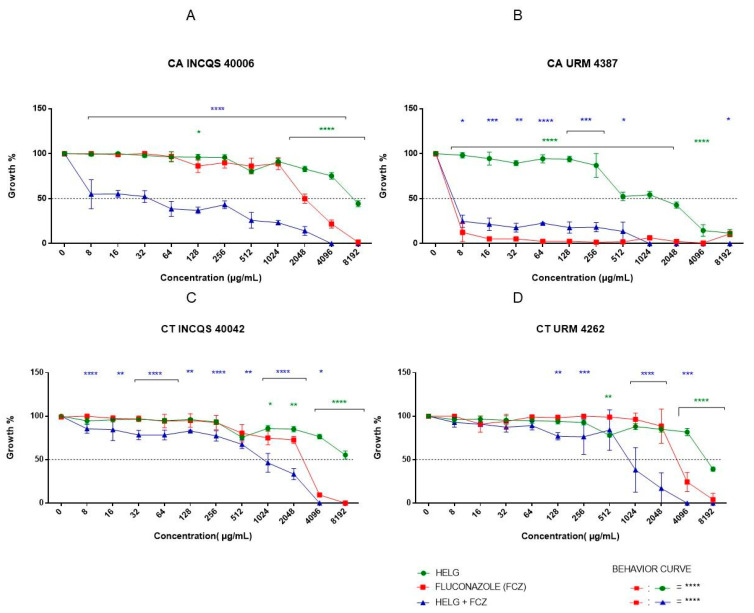
Cell viability curve of HELG alone and in combination with Fluconazole. *Candida* *albicans* INCQS 40006 (**A**), *Candida albicans* URM 4387 (**B**), *Candida tropicalis* INCQS 40042 (**C**) and *Candida tropicalis* URM 4262 (**D**). Statistical significance was determined considering the difference in relation to fluconazole, **** = *p* < 0.0001; *** = *p* < 0.001; ** = *p* < 0.01; * = *p* < 0.1.

The cell viability curves, which show the intrinsic activity of the aqueous (Figure 2) and hydroethanolic (Figure 3) extracts, demonstrated that both extracts presented weak antifungal activity, inhibiting only the growth of *C. albicans* URM 4387 and *C. tropicalis* INCQS 40042 at concentrations above 8.192 μg/mL. The pharmacological control fluconazole (FCZ) inhibited the growth of almost all strains from 8.192 μg/mL, except for the *C. albicans* URM 4387 strains, which showed higher sensitivity to the antifungal drug (64 μg/mL).

The minimum fungicidal concentration (MFC) was defined as the lowest concentration capable of fully preventing fungal growth. However, none of the tested substances (extracts and fluconazole) presented an MFC value comprehended in the concentration range adopted in this study (MFC ≥ 16.384 μg/mL). While the isolated extracts presented fungistatic effects, the combination of the hydroethanolic extract with fluconazole resulted in a fungicide effect against *C. albicans* URM 4387 (4096 μg/mL) and both *C. tropicalis* strains (2048 μg/mL).

To evaluate the ability of the extracts to potentiate the antifungal activity of fluconazole, the antifungal drug was serially diluted in the presence of a subinhibitory concentration of the extracts (MC/16 = 1024 μg/mL), considering the matrix concentration as better detailed below. It was demonstrated that extracts potentiated the effects of fluconazole in the standard strain of *C. albicans* and the clinical isolate of *C. tropicalis*. The tests with the URM 4387 strain of *C. albicans* showed that the aqueous extract did not change the inhibitory activity of fluconazole, while the hydroethanolic extract antagonized its effects.

The antifungal activity of the extracts and fluconazole was also represented in terms of their half-maximal inhibitory concentrations (IC_50_), as shown in Table 2. As observed, both extracts presented the most promising antifungal effects (lowest IC_50_ values) when tested against the URM 4387 strain of *C. albicans*. Curiously, the extracts potentiated (reduced the IC_50_) the antifungal activity of fluconazole against the other strain, but not against this clinical isolate.

#### 2.2.2. Effects of *G. hirsutum* Extracts on Biofilm Production by *Candida* Strains

**Figure 4:**
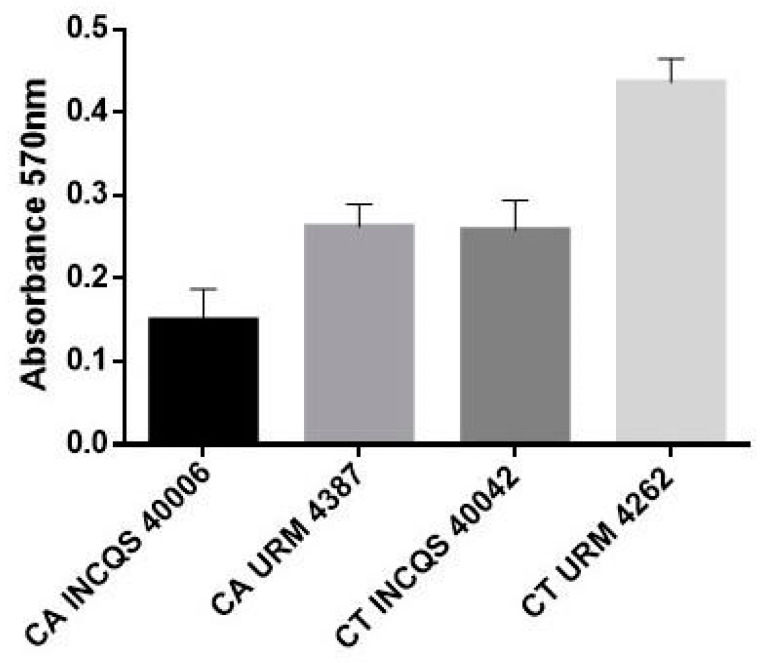
Biofilm formation by different *Candida* strains. The results are expressed as biofilm staining intensity read at 595 nm *C. tropicalis* INCQS 40042—moderate; *C. albicans* INCQS 40006; *C. tropicalis* URM 4262; *C. albicans* URM 4387. INCQS—National Institute for Quality Control in Health; URM—University Recife Mycology.

**Figure 5:**
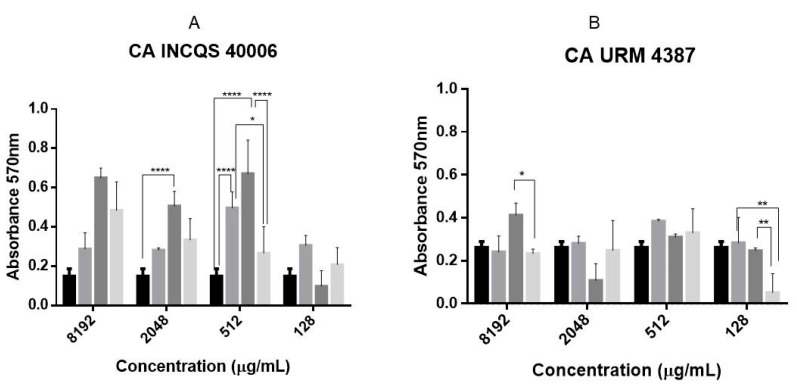

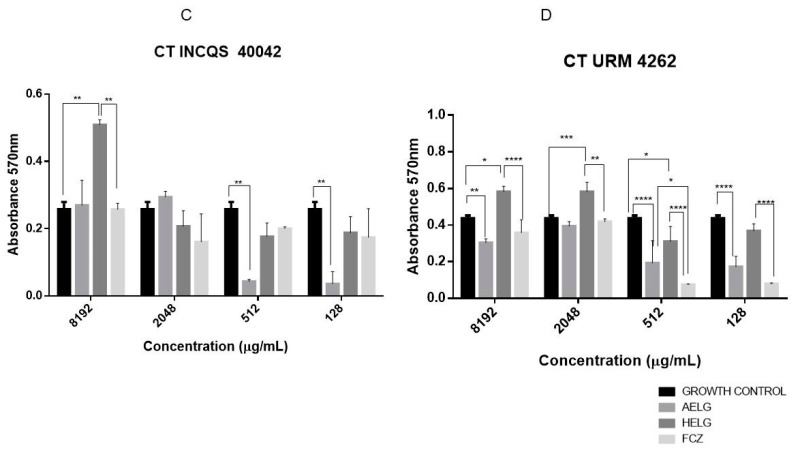
Effects of *G. hirsutum* extracts and fluconazole against *Candida* *albicans* INCQS 40006 (**A**), *Candida albicans* URM 4387 (**B**), *Candida tropicalis* INCQS 40042 (**C**) and *Candida tropicalis* URM 4262 (**D**). Statistical significance was determined considering the difference in relation to growth control/fluconazole. **** = *p* < 0.0001; *** = *p* < 0.001; ** = *p* < 0.01; * = *p* < 0.1. AELG—Aqueous extract of the leaves of *G. hirsutum*. HELG—Hydroethanolic extract of the leaves of *G. hirsutum*.

Figure 4 shows the biofilm production capacity of the strains under study, revealing that biofilm production varies among the strains, with *C. tropicalis* URM 4262 demonstrating the higher production capacity. An analysis of the effects of the different in vitro treatments (Figure 5) revealed that the HELG stimulated biofilm production by standard strains of *C. albicans* and *C. tropicalis* and the clinical isolate of *C. albicans* at all concentrations analyzed. On the other hand, the aqueous extract significantly inhibited biofilm production by the *C. tropicalis* isolate at 512 and 128 μg/mL. However, the higher concentrations stimulated biofilm production, probably due to the higher concentration of nutrients such as proteins and polysaccharides, which could contribute to biomass growth [25]. However, the mechanisms underlying this concentration-influenced phenomenon remain to be further investigated.

#### 2.2.3. *G. hirsutum* Extracts Inhibit *Candida* spp. Morphological Changes

**Figure 6:**
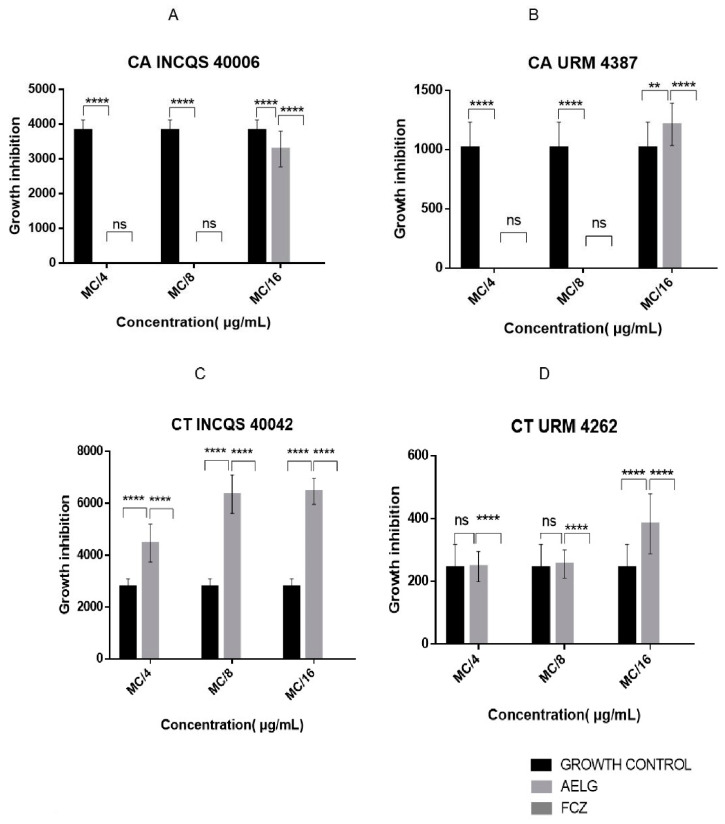
Effects of *G. hirsutum* aqueous extract and fluconazole on the morphological transition of *Candida* *albicans* INCQS 40006 (**A**), *Candida albicans* URM 4387 (**B**), *Candida tropicalis* INCQS 40042 (**C**) and *Candida tropicalis* URM 4262 (**D**). MC: Matrix Concentration (16.384 μg/mL); MC/4: 4.096 μg/mL, MC/8: 2.048 μg/mL, MC/16: 1.024 μg/mL; Growth inhibition—measured by micrometer-sized hyphae (µm); No bar—no hyphae growth. ** = *p* < 0.01, **** = *p* < 0.0001, ns = No Significant.

**Figure 7:**
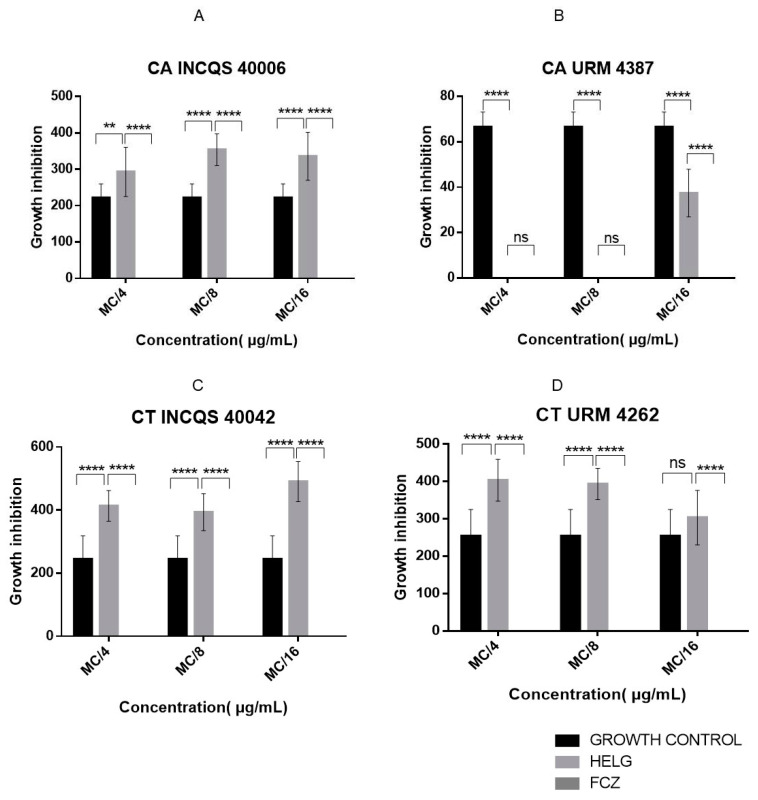
Effects of *G. hirsutum* hydroethanolic extract and fluconazole on the morphological transition of *Candida* *albicans* INCQS 40006 (**A**), *Candida albicans* URM 4387 (**B**), *Candida tropicalis* INCQS 40042 (**C**) and *Candida tropicalis* URM 4262 (**D**). MC: Matrix Concentration (16.384 μg/mL); MC/4: 4.096 μg/mL, MC/8: 2.048 μg/mL, MC/16: 1.024 μg/mL; Growth inhibition—measured by micrometer-sized hyphae (µm); No bar—no hyphae growth. ** = *p* < 0.01, **** = *p* < 0.0001, ns = No Significant.

To analyze the anti-pleomorphic effect of the extracts, we investigated their ability to inhibit the emission of hyphal formation by the fungal strains (Figure 6 and Figure 7). About the URM 4387 strain of *C. albicans*, it was found that both extracts fully inhibited hyphal growth at the relative concentrations of MC/4 and MC/8, while filament growth was stimulated at MC/16. The aqueous extract also showed significant inhibitory effects against the standard strain of *C. albicans* but had little impact on the morphological transition of *C. tropicalis clinical isolate*. Curiously, the standard strain of this species has its morphological transition significantly stimulated by all AELG concentrations. Concerning the hydroethanolic extract, except for the URM 4387 strain of *C. albicans*, significant hyphal growth was observed in all treatment conditions. Importantly, the morphological transition was fully inhibited by fluconazole, highlighting the effectiveness of this standard antifungal drug.

## 3. Discussion

The present research characterized the UPLC–QTOF-MS/MS profile and in vitro antifungal activity of the aqueous and hydroethanolic extracts of *G. hirsutum* L. (Malvaceae), a botanical species that is used in the treatment of urogenital tract infections in Brazilian folk medicine. The phytochemical analysis identified 15 of a total of 21 compounds of the samples, revealing that both extracts are predominantly constituted of phenolic compounds, such as epi-catechin, catechin, quercetin *O*-hexoside-pentoside, quercetin 3-*O*-glucoside, kaempferol *O*-rutinoside

Both extracts demonstrated moderate-to-weak intrinsic antifungal activity. Nevertheless, significant inhibition of fungal growth was observed against the standard strain of *C. albicans* and both *C. tropicalis* strains, which may be due to the presence of antimicrobial secondary metabolites such as flavonoids. Shetti et al. [26] reported that these compounds can accumulate in *G. hirsutum* specimens, causing their color to change from green to brown due to the expression of flavonoid-related genes. This class of compounds acts mainly as phytoalexins and photoprotectors, modulating the transport of the phytohormone auxin, which influences plant structure and function. In this context, it has been demonstrated that catechin is responsible for increasing the antifungal resistance of *G. hirsutum* to the fungus *Verticilium dahlia* [27], which may indicate a role of *G. hirsutum* flavonoids as antifungal agents.

Research conducted with herbalists from popular markets in the South of the Republic of Benin cited the use of decoction prepared from the leaves of *G. hirsutum* for the topical treatment of Candidiasis [28]. Studies have demonstrated the ethanolic extracts of the Malvaceae species *Guazuma ulmifolia* Lam. and *Luehea paniculata* Mart. & Zucc. demonstrated a potentiating effect when combined with the antifungal drug fluconazole against *C. tropicalis* and *C. albicans*. The authors have identified the presence of catechin, chlorogenic acid, rutin, and quercetin [29,30] compounds also identified in the extracts of *G. hirsutum* L., indicating a possible relation between the chemical content and biological activity of these species. Accordingly, a chloroformic extract obtained from *Sida cordifolia* (Malvaceae) showed antifungal activity against *C. albicans*, possibly due to the presence of flavonoids [31,32].

In addition to showing some intrinsic antifungal activity, *G. hirsutum* L. extracts were found to significantly enhance the activity of fluconazole against the standard and clinical isolates of *Candida* yeasts, which was observed through the reduction of the inhibitory concentrations of the standard antifungal drug when combined with a subinhibitory concentration of the extracts. Importantly, combined therapy has the benefit to improve the effectiveness of the treatment allied to lower doses, which can significantly reduce toxicity [33].

*Candida albicans*, followed by *C. tropicalis*, is the most important causative agent of opportunistic infections [34] in immunocompromised patients, which are more susceptible to the virulence arsenal of *Candida* yeasts, among which biofilm production and morphological change (emission of pseudohyphae and hyphae) are highlighted. In addition, these virulence mechanisms are associated with increased resistance to antifungal drugs such as azoles, placing *Candida* infections as an important public health concern [2].

Among the yeasts included in this study, the URM 4262 strain of *C. tropicalis* demonstrated the highest (strong) biofilm-producing potential, while the other strains showed moderate activity. These findings differ from those observed by [35], who found that *C. albicans* are better biofilm producers than non-albicans strains. On the other hand, previous research has corroborated the here demonstrated evidence that *C. tropicalis* are among the main biofilm producers of its genus [36].

Biofilm production by *Candida* strains contributes to antifungal resistance through different mechanisms, including by affecting cell density, changing the matrix constitution, promoting nutrient limitation, inducing the expression of the efflux pump and other resistance genes, as well by producing molecules that regulate cell morphology and biofilm maturation [37,38].

Understanding the resistance mechanisms associated with biofilms production may significantly contribute to the development of novel antifungal therapies. In this context, some compounds of natural products have been identified as promising drug candidates in an antimicrobial resistance context. Additionally, due to the novelty of many of these structures, secondary metabolites may represent promising alternatives for the treatment of fungal infections in immunocompromised patients [2,39].

Importantly, the present research demonstrated that *G. hirsutum* extracts showed a strain-selective and extract type-dependent capacity of inducing biofilm eradication, and therefore, further studies investigating the mechanisms underlying this phenomenon may significantly contribute to the targeted development of antibiofilm agents from this species [40].

Yeasts of the genus *Candida* can produce mycelium, a virulence factor that contributes to the maintenance of biofilms. In addition, these microorganisms can undergo a morphological transition from yeast to pseudohyphae and hyphae, which also represents an important virulence factor for opportunistic fungi [41]. Previous studies have demonstrated that flavonoids such as quercetin, quercitrin, isoquercetin, and rutin are capable of downregulating the expression of the CDR1 and ERG11 genes, which are significantly involved in the resistance of *C. albicans* by regulating biofilm production, hyphae growth, and many other virulence mechanisms [42]. From a clinical point of view, hyphae growth is an important mechanism used by the yeasts to penetrate the host tissues. Additionally, dimorphism is accompanied by changes in cell surface antigens, which represents a significant immune evasion mechanism. Thus, inhibiting fungal pleomorphism can significantly reduce the pathogenicity of yeasts [43].

While the UPLC–QTOF-MS/MS analysis allowed the identification of most secondary metabolites, the identification of some constituents was not possible. Nevertheless, in light of the specialized literature, it is suggested that the biological effects of the extracts can be attributed to the presence of phenolic compounds (especially flavonoids) in the extracts. Flavonoids are an extensive group of plant secondary metabolites with proven antifungal effects against *C. albicans*, *C. tropicalis*, and other fungal strains [44]. Compounds such as catechin, epi-catechin, quercetin, rutin, kaempferol, which were identified in the extracts, showed antifungal activity against *C. albicans* and *C. tropicalis* [45]. Of note, catechin, quercetin, and kaempferol derivatives were found to inhibit morphological transition, in addition to potentiating fluconazole activity against *Candida* yeasts [45,46]. Therefore, the development of further studies investigating the effectiveness of isolated phenolic compounds identified in the extracts would significantly contribute to understanding the anti-*Candida* potential of *G. hirsutum.*

## 4. Materials and Methods

### 4.1. Botanical Material

*Gossypium hirsutum* specimens were collected in a rural area of the municipality of Araripe, Ceará, Brazil (coordinates: 7°12′12.5″ S 40°01′10.2″ W; 7°12′23.1″ S 40°00′50.7″ W; 7°13′38.5″ S 39°59′44.5″ W). The collection took place in April 2019, from 7:30 to 9:00 in the morning. The plant material was sent to the laboratory, cleaned, and weighed. A voucher specimen was registered in the Herbarium of the Regional University of Cariri—URCA under registry number 65.2019.

### 4.2. Extract Preparation

For the preparation of the extracts, a total of 1172.3 g of fresh leaves were cut and dried at room temperature. Amounts of 512.8 g and 654.5 g were used in the preparation of the aqueous and hydroethanolic extracts, respectively. Then, distilled water or 70% ethanol was added to the corresponding sample, which was kept protected from light and air [47]. After 72 h, the extracts were filtered and taken to a spray-drying (130 °C, flow rate 0.5 L/h, blower control 1.95, outlet temperature 84.6, flow meter 40 L/min air, particles with one millimeter in diameter), producing crude extracts yielding 8380 and 5486 g, respectively.

### 4.3. Candida Strains

The clinical isolates (*Candida albicans* URM 4387 and *Candida tropicalis* URM 4262) were obtained from the Federal University of Pernambuco by the University Recife Mycologia (URM). The standard strains of *Candida albicans* INCQS 40006 (ATCC 10231), *Candida tropicalis* INCQS 40042 (ATCC 13803) were obtained from the National Institute for Quality Control in Health (INCQS, FIOCRUZ).

### 4.4. Chemical Characterization by UPLC–QTOF-MS/MS

The samples were filtered through PTFE syringe filters (0.2 μm pore, Millipore Millex, Sigma-Aldrich^®^, Darmstadt, Germany) and 5 μL of each sample were injected into the ultra-performance liquid chromatography coupled to quadrupole/time of flight system (UPLC-QTOF-MS/MS) (Waters Co., Milford, MA, USA) equipped with an ACQUITY UPLC BEH column (150 × 2.1 mm, 1.7 µm, Waters Co.) set to 40 °C. The binary gradient elution system consisted of 0.1% formic acid in water (A) and 0.1% formic acid in acetonitrile (B), with a linear gradient from 2 to 95% B (0–15 min), 100% (15.01–17 min), 2% (17.01–21 min) and a flow rate of 0.4 mL.min−1. The samples were analyzed through an electrospray ionization interface (ESI) in the negative ionization mode. The desolvation gas used was nitrogen and set to 350 °C with a flow rate of 500 L/h. The capillary and cone voltages were adjusted to 2.6 kV and 0.5 V, respectively. Mass accuracy and reproducibility were maintained by infusing lock mass (leucine-enkephalin, 0.2 ng/uL; [M-H]^−^ ion at *m*/*z* 556.2771). MS data were collected for *m*/*z* values in the range of 110–1180 Da with a scan time of 0.1 over an analysis time of 19 min. The accurate mass and molecular formula assignments were obtained with the MassLynx 4.1 software (Waters MS Technologies).

### 4.5. Anti-Candida Activities

#### 4.5.1. Cell Viability Curve, IC_50_ and Minimum Fungal Concentration (MFC)

The strains were maintained in Sabouraud Dextrose Agar (SDA, KASVI) medium and incubated for 24 h at 37 °C. An initial suspension was subsequently prepared in 5 mL of sterile saline solution (NaCl, 0.85% saline) and its turbidity was visually adjusted according to the 0.5 value of the MacFarland scale, providing a standard concentration of yeasts ranging from 1 × 10^6^ to 5 × 10^6^ cells per mL.

An initial solution was prepared by adding 1350 μL of doubly concentrated SDA and 150 μL of the fungal strain inoculum (corresponding to 10% of the total solution). Then, 150 μL of this solution was removed and added to each well on a microdilution plate. After this, the wells were filled with 100 μL of extract solution at concentrations ranging from 8192 to 8 μg/mL, and the plates were incubated at 37 °C. After 24 h, the readings were performed at 630 nm in a spectrophotometer (Thermoplate^®^). The optical density values were used to build the cell viability curve and calculate the IC_50_. Diluent (0.9% NaCl) sterility and growth controls were also used [48]. All tests were performed in quadruplicate.

For MFC determination, a sterile rod was inserted into each well of the microdilution plate, which was homogenized and subcultured in a Petri dish containing SDA. The plates were incubated at 37 °C, and readings were taken after 24 h, by observing the growth of *Candida* colonies [49]. The MFC was defined as the lowest concentration capable of fully preventing fungal colony growth. Then, the combined activity and morphological transition were evaluated by using MFC-based subinhibitory concentrations. For the treatments whose MFC could not be determined, the Matrix Concentration (MC) was used.

#### 4.5.2. Enhancement of Fluconazole Antifungal Activity

To evaluate the ability of the extracts to potentiate the antifungal activity of fluconazole, the antifungal drug was serially diluted in the presence of a subinhibitory concentration of the extracts (MC/16 = 1024 μg/mL) [33]. Briefly, the plates were numerically filled by adding 100 μL of a solution containing the extract, culture medium (SDB), and 150 μL of the microbial suspension (corresponding to 10% of the solution). Growth and dilution controls were prepared. Thereafter, serial dilutions of fluconazole were performed as previously described, and 100 μL of the drug were added to each well on the plate. The plates were incubated at 37 °C for 24 h, and the readings were carried out as previously described.

### 4.6. Evaluation of Biofilm Eradication by Candida *spp.*

To prepare the treatment solutions, 0.15 g of each extract was dissolved in 1 mL of dimethyl sulfoxide (DMSO, Merck, Darmstadt, Germany) and diluted to concentrations ranging from 16.384 μg/mL to 128 μg/mL. Fluconazole (Pfizer) was used as a reference antifungal drug at concentrations varying between 64 μg/mL and 0.5 μg/mL.

The qualitative evaluation of biofilm formation capacity was performed using the visual method adapted from [50]. After seeding the isolates in the Sabouraud agar medium and preparing the suspensions as described above, 20 μL of inoculum and 180 μL of liquid Sabouraud medium were added to the wells on the microplate and kept at 35 °C for 72 h without shaking. The contents were then aspirated and the wells were washed with distilled sterile water and a Fuchsin dye (QEEL-Specialized Chemistry Erich Ltd. São Paulo, Brazil).

The staining intensity was evaluated visually and classified as follows: (1) Strongly stained: when the biofilm was intensely stained allowing the correct determination of the contour of the areas containing the biofilm; (2) Average staining: when the biofilm was stained more weakly but still allowed the determination of the contour of the areas containing the biofilm; (3) Poor staining: when the biofilm was not stained, making it impossible to distinguish the color of the plate and the characteristic color of the dye. According to the staining intensity, the biofilm production activity was classified as strong, moderate, and weak.

The induction of biofilm formation and the in vitro treatments were performed according to previous reports [36,51]. Briefly, 20 μL of the suspensions and 180 μL of YPD (Yeast Extract-Peptone-Dextrose) medium were transferred to the wells on a 96-well plate. The plates were incubated at 37 °C for 72 h. Following the incubation time, the wells were carefully aspirated and washed twice with 200 μL of PBS buffer. The prewashed wells were stained with 110 μL of 0.4% aqueous violet crystal solution for 45 min. Thereafter, they were washed three times with 200 μL of ultrapure sterile water (Milli-Q) and the biofilm was discolored using 200 μL ethanol for 45 min. Later, 100 μL of the well content was transferred to a new microplate and biofilm formation was evaluated using the optical density difference between the biofilm formed and the control well, by reading the absorbance in a spectrophotometer adjusted with a wavelength of 595 nm. Each strain was tested three times, and the absorbance values of the control wells were subtracted from the tested wells to minimize interference [36].

To analyze the impact of the extracts, following 48 h of biofilm production, the wells were filled with 200 μL of either the *G. hirsutum* L. or fluconazole following the serial dilution using RPMI 1640 medium to achieve each concentration as previously reported. Untreated wells and biofilm-free wells were included as positive and negative controls, respectively. The microdilution plates were incubated for 72 h at 35 °C. After this period, the biofilm was quantified as previously described [51].

### 4.7. Effects of G. hirsutum Extracts on the Morphological Transition of Candida Yeasts

A sterile microscope slide was filled with 3 μL of depleted Potato Dextrose Agar (PDA), to which the extracts were added at MC-based concentrations (MC/4, MC/8, and MC/16) and mounted in humid chambers. A yeast loop was removed from a previously inoculated plate and two parallel grooves were made in the solidified medium, over which a sterile coverslip was placed. The chambers were incubated and visualized under an optical microscope (AXIO IMAGER M2-3525001980—ZEISS—Jena, Germany) using a 20× objective after an incubation period of 24 h at 37 °C. The emission of filament was verified, and the images were captured. Growth and treatment (fluconazole) controls were also applied. Thereafter, the images were analyzed by measuring the total length of the inoculum striae and the length of hyphal or pseudohyphal extensions in µm using the Zen 2.0 software, ZEISS—Jena, Germany [52].

### 4.8. Statistical Analysis

Data derived from biofilm production and the cell viability curves were analyzed using the arithmetic mean from triplicates or quadruplicates, respectively, for each tested concentration. The differences were analyzed using two-way ANOVA with Bonferroni’s post hoc test. The average hyphal length was calculated and analyzed by one-way ANOVA followed by Bonferroni’s correction for multiple comparisons. These analyses were performed using the GraphPad Prism software version 5.0. Statistical significance was considered when *p* < 0.05.

## 5. Conclusions

Both aqueous and hydroethanolic extracts of *G. hirsutum* are characterized by the known bioactive secondary metabolites such as catechin and epicatechin, showing a predominance of phenolic compounds. Nevertheless, the extracts had quantitative differences in their constituents as the hydroethanolic extract demonstrated higher relative intensity for coumaroylglucaric acid, catechin, chlorogenic acid, and epicatechin, while the aqueous extract showed higher relative intensity for quercetin *O*-hexoside-pentoside.

While both extracts showed weak-to-moderate intrinsic antifungal activity, they were found to downregulate important virulence factors of both standard and clinical isolates of *Candida* spp. The aqueous was found to enhance the antifungal activity of fluconazole against *C. albicans* INCQS 40006 and *C*. *tropicalis* URM 4262, and the hydroethanolic extract combined with fluconazole showed potentiated inhibitory effects on the growth of *C. albicans* INCQS 40006. *G. hirsutum* extracts showed a strain-selective and extract type-dependent capacity of inducing biofilm eradication, highlighting the effectiveness of the aqueous extract against the standard and clinical isolates of *C. tropicalis*, especially at lower concentrations. However, a significant increase in biofilm content was observed for most experimental conditions; further studies investigating the mechanisms underlying this phenomenon may significantly contribute to the targeted development of antibiofilm agents from this species. Finally, the aqueous extract inhibited *C. albicans* hyphal growth on both standard and clinical isolate strains, while the hydroethanolic extract inhibited hyphal growth only for this clinical isolate.

In conclusion, the present research found that *G. hirsutum* has the potential to enhance the virulence of standard and clinical isolates of *Candida albicans* and *Candida tropicalis* in vitro. However, such phenomenon was found to be highly affected by experimental variables including type and concentration of extracts as well as the species and strain of *Candida*. Therefore, further research is strongly recommended to better characterize the antifungal properties of this species about its chemical constituents and their mechanisms of action.

## Figures and Tables

**Table 1:** Chemical profile of the aqueous and hydroethanolic extracts obtained from the leaves of *Gossypium hirsutum* L.

Peak	Rt Min	[M-H]^−^ Observed	[M-H]^−^ Calculated	Product Ions (MS/MS)	Empirical Formula	Ppm Error	Putative Name	Ref.	A ^a^	H ^a^
**1**	1.28	191.0190	191.0192	111	C_6_H_7_O_7_	−1.0	Citric acid *	[19]	+	+
**2**	1.88	371.0623	371.0614	209, 191	C_15_H_15_O_11_	2.4	Caffeoylglucaric acid	[18]	+	+
**3**	2.17	371.0624	371.0614	209, 191	C_15_H_15_O_11_	2.7	Caffeoylglucaric acid	[18]	+	+
**4**	2.25	371.0613	371.0614	209, 191	C_15_H_15_O_11_	−0.3	Caffeoyglucaric acid	[18]	+	+
**5**	2.48	371.0606	371.0614	209, 191	C_15_H_15_O_11_	−2.2	Caffeoylglucaric acid	[18]	+	+
**6**	2.67	355.0653	355.0665	209, 191	C_15_H_15_O_10_	−3.4	Coumaroylglucaric acid	[18]	+	++
**7**	3.02	289.0722	289.0712	-	C_15_H_13_O_6_	3.5	Catechin *	[20]	+	+++
**8**	3.04	353.0874	353.0873	191	C_16_H_17_O_9_	0.3	Chlorogenic acid *	[21]	+	+++
**9**	3.16	163.0399	163.0395	119	C_9_H_7_O_3_	2.5	*p*-coumaric acid *	[21]	+	+
**10**	3.32	179.0336	179.0344	135	C_9_H_7_O_4_	4.5	Caffeic acid	[20]	+	+
**11**	3.52	289.0718	289.0712	-	C_15_H_13_O_6_	2.1	Epi-catechin *	Std	+	+++
**12**	3.56	469.1721	469.1710	359	C_22_H_29_O_11_	2.3	Unknown	−	+	+
**13**	3.70	467.1564	467.1553	359	C_22_H_27_O_11_	2.4	Unknown	−	+	+
**14**	3.81	517.1037	517.1041	241	C_17_H_25_O_18_	−0.8	Unknown	−	+	+
**15**	4.11	595.1310	595.1299	301, 300	C_26_H_27_O_16_	1.8	Quercetin *O*-hexoside-pentoside	[22]	++	+
**16**	4.27	609.1444	609.1456	301, 300	C_27_H_29_O_16_	−2.0	Rutin *	[20]	+	+
**17**	4.41	463.0869	463.0877	301, 300	C_21_H_19_O_12_	−1.7	Quercetin 3-*O*-glucoside *	[13,23]	+	+
**18**	4.62	593.1512	593.1506	285, 255	C_27_H_29_O_15_	1.0	Kaempferol *O*-rutinoside	[24]	+	+
**19**	4.97	451.1646	451.1663	96.9579	C_15_H_31_O_15_	−3.8	Unknown	−	+	+
**20**	5.66	501.1093	501.1092	96.9587	C_17_H_25_O_17_	0.2	Unknown	−	+	+
**21**	5.84	453.1818	453.1819	96.9605	C_15_H_33_O_15_	−0.2	Unknown	−	+	+

Rt: retention time; A ^a^: aqueous extract; H ^a^: hydroethanolic extract. +, ++, +++ Relative intensity ratio shows the higher compound concentration in each extract. * STD, comparison with an authentic standard.

**Table 2:** IC_50_ of the AELG and HELG against different strains of *Candida*.

	CA INCQS 40006	CA URM 4387	CT INCQS 40042	CT URM 4262
**AELG**	2257	317.2	2214	2986
**AELG + FCZ**	62.76	2.344	819.8	628.5
**HELG**	8865	1165	NI	8567
**HELG + FCZ**	29.12	2476	701.1	788.0
**FLUCONAZOLE**	2125	0.036	2476	3196

NI: Not identified.

## Data Availability

Not applicable.
